# Functional defects in hiPSCs-derived cardiomyocytes from patients with a PLEKHM2-mutation associated with dilated cardiomyopathy and left ventricular non-compaction

**DOI:** 10.1186/s40659-023-00442-5

**Published:** 2023-06-23

**Authors:** Nataly Korover, Sharon Etzion, Alexander Cherniak, Tatiana Rabinski, Aviva Levitas, Yoram Etzion, Rivka Ofir, Ruti Parvari, Smadar Cohen

**Affiliations:** 1 Avram and Stella Goldstein-Goren Department of Biotechnology Engineering, Ben-Gurion University of the Negev, 84105 Beer-Sheva, Israel; 2 Regenerative Medicine & Stem Cell Research Center, Ben-Gurion University of the Negev, 84105 Beer-Sheva, Israel; 3 Department of Pediatric Cardiology, Soroka University Medical Center and Faculty of Health Sciences, Ben-Gurion University of the Negev, 84105 Beer-Sheva, Israel; 4 Department of Physiology and Cell Biology, Faculty of Health Sciences, Ben-Gurion University of the Negev, 84105 Beer-Sheva, Israel; 5 Dead Sea & Arava Science Center, 8691000 Masada, Israel; 6 Department of Microbiology, Immunology and Genetics, Faculty of Health Sciences, Ben-Gurion University of the Negev, 84105 Beer-Sheva, Israel; 7 National Institute for Biotechnology in the Negev, Ben-Gurion University of the Negev, Beer-Sheva, Israel; 8 Ilse Katz Institute for Nanoscale Science and Technology, Ben-Gurion University of the Negev, 84105 Beer-Sheva, Israel

**Keywords:** Autophagy, Induced pluripotent stem cell, Cardiomyocytes, MRI, Cardiomyopathy, Fibrosis

## Abstract

Dilated cardiomyopathy (DCM) is a primary myocardial disease, leading to heart failure and excessive risk of sudden cardiac death with rather poorly understood pathophysiology. In 2015, Parvari's group identified a recessive mutation in the autophagy regulator, PLEKHM2 gene, in a family with severe recessive DCM and left ventricular non-compaction (LVNC). Fibroblasts isolated from these patients exhibited abnormal subcellular distribution of endosomes, Golgi apparatus, lysosomes and had impaired autophagy flux. To better understand the effect of mutated PLEKHM2 on cardiac tissue, we generated and characterized induced pluripotent stem cells-derived cardiomyocytes (iPSC-CMs) from two patients and a healthy control from the same family. The patient iPSC-CMs showed low expression levels of genes encoding for contractile functional proteins (α and β-myosin heavy chains and 2v and 2a-myosin light chains), structural proteins integral to heart contraction (Troponin C, T and I) and proteins participating in Ca^2+^ pumping action (SERCA2 and Calsequestrin 2) compared to their levels in control iPSC-derived CMs. Furthermore, the sarcomeres of the patient iPSC-CMs were less oriented and aligned compared to control cells and generated slowly beating foci with lower intracellular calcium amplitude and abnormal calcium transient kinetics, measured by IonOptix system and MuscleMotion software. Autophagy in patient’s iPSC-CMs was impaired as determined from a decrease in the accumulation of autophagosomes in response to chloroquine and rapamycin treatment, compared to control iPSC-CMs. Impairment in autophagy together with the deficiency in the expression of NKX2.5, MHC, MLC, Troponins and CASQ2 genes, which are related to contraction-relaxation coupling and intracellular Ca^2+^ signaling, may contribute to the defective function of the patient CMs and possibly affect cell maturation and cardiac failure with time.

**Supplementary Information:**

The online version contains supplementary material available at 10.1186/s40659-023-00442-5.

## Introduction

Cardiomyopathies represent a heterogeneous group of cardiac diseases with structural and functional changes in the myocardium that can cause heart failure (HF) and death [1, 2]. Among this group of diseases are dilated cardiomyopathy (DCM) and left ventricular non compaction (LVNC) cardiomyopathy. DCM is characterized by dilatation of the left or both ventricles that is not explained by abnormal loading conditions (e.g., hypertension, valvular diseases) or coronary artery disease sufficient to cause global systolic impairment [1]. LVNC is a congenital cardiomyopathy characterized by a distinctive spongy appearance of the myocardium, due to increased trabeculation and deep inter-trabecular recesses in hypertrophied and hypokinetic segments of the left ventricle that communicate with the left ventricular cavity [3, 4]. LVNC is thought to represent failure in maturation of ventricular cardiomyocytes, which become compacted myocardium [5]. This defect exhibits variability in presentation from neonates to adulthood, but appears to have a congenital etiology. LVNC and DCM may occur concurrently [6]. The causes for DCM/LVNC are manifold and can be classified as familial, i.e., genetic, as well as non-familial (acquired) forms due to infections, toxic substances, or autoimmune diseases [4, 7]. Autosomal dominant inheritance is the most common inheritance form [8], presenting usually in the second or third decade of life [9, 10]. Autosomal-recessive mutations leading to DCM are far less common than the dominant form. More than 40 mutated genes are known today to be associated with cardiomyopathy and mostly code for ion channels, sarcomeres, Z-discs, nuclear proteins, and desmosomes [11–17].

In the last decade, a large body of evidence has indicated alterations of autophagy in a wide range of cardiac diseases, including cardiomyopathies [18, 19]. The role of autophagy in inherited cardiomyopathies may be particularly important because in some cases mutant/misfolded protein may be the cause of the disease. Yet, only a few forms of inherited cardiomyopathies are known to be associated with a defect in autophagy. Muhammad et al. [20] described a large Bedouin family presenting with a severe recessive DCM and LVNC and harboring a mutation in pleckstrin-homology-domain-containing family member 2 (PLEKHM2*)* gene (cDNA2156_2157delAG), encoding pleckstrin homology domain-containing family M member 2, an autophagy regulator [20].

The PLEKHM2 protein (sometimes referred to as SKIP) has been found vital for endocytotic trafficking. The small GTPase Arl8, a member of the Arf-like (Arl) family of proteins, bound to GTP, is recruited to the lysosomal membrane in a complex with PLEKHM2 and kinesin, which binds to microtubules [21, 22]. This complex controls lysosome positioning in the cells [23]. Lysosomal positioning influences autophagosome formation and autophagosome-lysosome fusion rates, and thus controls autophagic flux by acting both at the initiation and termination stages of the process [21]. In the DCM/LVNC patients [20], the recessive mutation is identified as a frameshift eliminating half of the amino acids of the protein at its carboxy-terminus, and a deletion of 30 highly conserved amino acids eliminating the pleckstrin-homology domain of PLEKHM2. Primary fibroblasts from these patients exhibited abnormal subcellular distribution of endosomes marked by RAB5, RAB7 and RAB9, abnormal lysosome localization and impaired autophagic flux [20]. Whether and how this mutation affects cardiomyocytes (CMs) in these patients remains unknown.

Herein, we developed a novel "disease in a dish" model from PLEKHM2-mutated patient cells to investigate the mechanism behind this disease phenotype. We generated and characterized induced pluripotent stem cell (iPSC)-derived CMs from two patients and a healthy control from the same family. We studied the morphology and function of the DCM-PSCs-CMs, recoded their contractility, assessed Ca^2+^ handling and quantified the autophagy flux in response to chloroquine and rapamycin treatment. Our findings indicate that the PLEKHM2 mutation leading to DCM-LVNC is associated with impaired autophagy in CMs and that this biochemical abnormality may play a critical role in the cardiac dysfunction and myocardial tissue loss in these patients.

## Materials and methods

All of the protocols for this study were approved by the Ben-Gurion University Human subjects Research Institutional Review Board.

### Image acquisition

Cardiac MRI (CMR) scans were performed at Soroka University Medical Center using a 1.5-Tesla Philips Achieva scanner (Philips Medical Systems, Best, The Netherlands). Imaging was performed with the patient in the supine position, using a 32-element cardiac phased-array coil and ECG gating. All acquisitions were obtained during breath holding in expiration. Localizing scans were followed by breath-hold cine acquisitions in the ventricular long-axis, 2-chamber, and horizontal long-axis planes to ensure accurate planning of the LV short-axis orientation. Multiple-slice data sets, parallel to the mitral valve, covering the heart in 10–12 short-axis slices, were acquired. A steady state free precession (SSFP) pulse sequence was performed (TR 3.34 ms, TE 1.67 ms, flip angle 55°, bandwidth 1042 Hz/pixel, acquisition matrix 192 × 163, FOV 360 × 288 mm, half Fourier acquisition, 6-mm slice thickness, 4-mm interslice gap, 18 phases/cardiac cycle, with the slices acquired per 10- to 12-s breath hold). Phase-sensitive inversion recovery (PSIR) images in long axis, four chambers and short-axis orientations were used to study late enhancement 10 min following gadolinium injection.

### Materials

Matrigel^®^-coated plates were from BD Biosciences (San Jose, CA). ROCK inhibitor (Y-27632) was from R&D Systems, Minneapolis, MN; CHIR99021 and IWP2 were purchased from Tocris, United Kingdom. Cell culture reagents: NutriStem^®^ hESC XF Culture Media, Dulbecco’s modified Eagle’s medium (DMEM), l-glutamine, penicillin/streptomycin, heat inactivated Fetal Bovine Serum (FBS), CryoStem™ Freezing Medium, MEM Non-Essential Amino Acids Solution were from Biological Industries (Kibbutz Beit-Haemek, Israel). B27 supplemented with and without insulin, Roswell Park Memorial Institute (RPMI) 1640 medium, StemPro^®^ Accutase® Cell Dissociation Reagent, 2-Mercaptoethanol (50 mM) and TrypLE TM were from GIBCO (Gaithersburg, MD). CytoTune™-iPS 2.0 Sendai Reprogramming Kit and bFGF Recombinant Human Protein were from Invitrogen (California, USA). CYTO-ID^®^ Autophagy Detection Kit was from ENZO Life Sciences (New York, USA). Other reagents, unless specified otherwise, were from Sigma-Aldrich (Rechovot, Israel). All reagents were of analytical grade.

### Cell cultures

Induced pluripotent stem cells (iPSCs**)** were generated from adult somatic cells of patients with recessive mutated PLEKHM2 (termed DCM-R and DCM-O), and from a family member carrying mutation only on one allele (termed DCM-C). All primary fibroblast lines were thawed and grown in cell culture flasks (25-cm^2^) in fibroblast medium [(10–15% FBS, 1% MEM-NEAA, 10 mM and 0.1–0.2% 2-Mercaptoethanol, 50 mM in high-glucose DMEM (P_0_)].

### Reprogramming of human skin fibroblasts with Sendai virus vectors

Biopsies of skin (DCM-C and DCM-R) and of internal connective tissue taken while performing a surgery for pacemaker replacement (DCM-O) were obtained from three studied participants. The primary cells have a different growth rate, therefore, the day before transduction, the cells (termed P_2_) were seeded at different concentrations on 6 well plates: DCM-C 2*10^5^ cells/w, DCM-R 2.5*10^5^ cells/w and DCM-O 3*10^5^ cells/w in fibroblast medium.

Reprogramming of the cells was performed using Sendai virus (SeV) CytoTune™ 2.0 Sendai reprogramming kit (expressing the transcription factors Oct, Sox2, Klf4 and c-Myc), according to the manufacturer’s protocol recommendations [24]. On the day of transduction, 1 mL of fibroblast medium, with the calculated volumes of each of the three CytoTune™ 2.0 Sendai tubes, was added to the cells. The cells were incubated 1–2 days at 37 °C. 48 h post transduction, and every other day, the medium was replaced with fresh medium. On day 7, the fibroblast cells were harvested using 1 mL per well of TrypLE™ and plated onto a feeder layer of irradiated mouse embryonic fibroblast (MEFs) coated 6 well plates and grown in MEF-conditioned medium. 24 h later, the medium was switched to NutriStem^®^ hESC XF medium supplemented with 5 ng/mL bFGF, and thereafter replaced every two days. The typical undifferentiated iPSC colonies were picked up using a sterile tip, transferred onto prepared matrigel-coated 6- or 12-well culture plates for further expansion and grown with NutriStem^®^ hESC XF media. Passaging was done using mechanical or enzymatic (Accutase) treatment. Reprogrammed cell lines were monitored for pluripotency by flow cytometry analysis and immunofluorescent staining, using the markers TRA-1–60, SSEA-4 and Oct ¾.

### Genotype validation

Genomic DNA was extracted using a Puregene Cell Kit (Qiagen, Carlsbad, CA, USA). A 687 bp fragment of PLEKHM2 exon 12 containing the mutation site was amplified by PCR. The primer sequences are provided in Table 1. Sanger sequencing was performed using the NIBN DNA sequencing laboratory (Be’er Sheva, Israel).

**Table 1:** Primers of mutation sequencing used for genotype validation

Primers	Target	Forward/Reverse primer (5′-3′)
Targeted mutation sequencing	PLEKHM2 exon 12	Forward TTGTAGACGAGGCTGACTCTCA Reverse CAGAAACCACACCGTGACAT

### Spontaneous differentiation [Embryoid bodies (EBs) formation]

EBs were formed using the hanging drop method. This method provides uniform sizes of EBs by dispensing equal numbers of iPSCs in physically separated droplets of media suspended from the lid of a Petri dish. In order to form the droplets, medium was aspirated and the iPSC culture was washed with 3 mL per well of sterile PBS. 1 mL per well of TrypLE™ was added and cells were incubated at 37 °C until the edges of the colonies started to loosen and slightly fold up (2–3 min). 2 mL per well of NutriStem^®^ hESC XF Culture Medium was added and colonies were scraped and washed off with a 5 mL of PBS. IPSC suspension was diluted to a concentration of 1000 -2000 cells per 20 µL (20 µL/drop) in EBs medium (NutriStem^®^ hESC XF, bFGF 10 ng/mL, ROCK Inhibitor 5μM) and the droplets were put on a sterile lid of a dish. The lid was lifted, inverted and placed on top of the dish containing 5 ml of PBS. The dish was placed in the incubator for 2 days. After 2 days, the drops were picked up with the pipette and transferred to a low adherence bacterial Petri dishes. The plates were placed into the incubator for an additional 4 or 10 days with gradually adding of EB differentiation medium, for spontaneous or cardiac differentiation, respectively. For immunostaining, following incubation the EBs were transferred from the suspension tissue culture dish to the 12 well plates coated with 0.1% gelatin. 1 mL of differentiation medium (10% FBS, 1% Pen-Strep-Amphotericin Solution and 0.2% 2-Mercaptoethanol, 50 mM in high-glucose DMEM) was added to each well. The medium was changed the next day and then every other day.

### Flow cytometry analysis

For flow cytometry analysis of iPSCs and differentiated iPSC-CMs, the cells were dissociated into a single-cell suspension using TrypLE™ for 2–4 min (for iPSCs) or collagenase type II (95 U/mL) (for iPSC-CMs) for 15 min. The cells were fixed and permeabilized for intracellular staining with the Cytofix/Cytoperm kit (BD Pharmingen) following the manufacturer’s recommended protocol. The cells were immuno-stained with relevant antibodies dissolved in FACS buffer (PBS with 2% FBS, v/v) to detect intracellular and surface markers. Cell acquisition and analysis were performed using a FACS-Canto machine (BD Biosciences, San Jose, CA), utilizing Cellquest Pro software (BD Biosciences) and FlowJo 7.6.5. The primary antibodies used are: anti-TRA-1–60 (MAB4770, 1:50, R&D systems), anti-OCT4 (sc-5276, 1:100, Santa Cruz), anti-SSEA-4 (sc-21704, 1:100, Santa Cruz), anti-68 kDa Neurofilament (ab52989, 1:100, Abcam), anti-α-SMA (ab32575, 1:100, Abcam), anti-AFP (A00058.0025, Scytec), anti-cTNT (ab8295, 1:100, Abcam), anti-Vimentin (180052, 1:200, Thermo Fisher Scientific), anti-Human-CD90/Thy1 Alexa Fluor conjugated (FAB2067G, 1:20, R&D systems) and anti-Human-CD172s (SIRP alpha) APC conjugated (17-1729, 1:20, eBioscience). Secondary antibodies used: donkey anti-mouse Alexa 488 antibody (715-545-151, 1:250, Jackson), donkey anti-rabbit Alexa 488 antibody (711-545-152, 1:250, Jackson), donkey anti-goat Alexa 488 antibody (705-546-147, 1:250, Jackson), donkey anti-rabbit Alexa 647 antibody (711-605-152, 1:250, Jackson), and Alexa 488 Streptavidin (016-540-084, 1:2000, Jackson). 

### Teratoma assay

IPSCs were dissociated into a single cell suspension using TrypLE™ for 2–4 min. The pellet was re-suspended in NutriStem^®^ to a total volume of 50 μL and combined with 450 μL undiluted cold Matrigel. 2*10^6^ cells were injected under the skin of immunodeficient NOD SCID mice. Approximately 8–10 weeks post-injection, any visible tumors were dissected and fixed with 4% PFA, then were paraffin embedded, sectioned and stained with Hematoxylin and Eosin. The presence of tissue representatives of the three germ layers were visualized by confocal microscope.

### Immunostaining and confocal imaging

For immunofluorescence and confocal imaging, the monolayer cells were cultured for 24–72 h on ibidi slides (ibidi, Madison, WI) or cover glass coated with 0.1% gelatin or Matrigel^®^, depending on cell type. Then, the 2D monolayer culture was fixed in 4% (v/v) warm methanol-free formaldehyde in PBS for 7–10 min, washed in PBS with 1% BSA (PBS buffer) and permeabilized using Triton-X 100 [0.1% (v/v) in PBS buffer] for 1 h at RT. The samples were incubated overnight with anti-α-actinin antibodies (Clone EA-53, 1:300, Sigma) or anti-cTNT (ab8295, 1:100; Abcam) or anti-LC3B antibodies (NB100-2220, 1:200, Novus Biologicals), followed by 2 h incubation with Alexa 488-conjugated donkey anti-mouse antibodies (715-545-151, 1:250, Jackson) or Alexa 488-conjugated donkey anti-rabbit antibodies (711-545-152, 1:250, Jackson), Alexa-Fluor 546-conjugated phalloidin (A22283, 1:200, Life Technologies) for staining F-actin and VECTASHIELD Mounting Medium with DAPI (Vector Laboratories, Burlingame, CA) for nuclei detection. Imaging was performed with a Nikon C1si laser scanning confocal microscope (LSCM).

### Cardiac monolayer differentiation by small molecule supplementation

A small molecule monolayer differentiation modified protocol was used for cardiac differentiation [25, 26]. Undifferentiated iPSC clones were cultured on Matrigel^®^ with NutriStem^®^ hESC XF media, until they reached 80% confluence, and then passaged using Versene solution. Then, the cells were seeded at 8*10^5^ cells per well onto 12 well plates coated with double concentration of Matrigel^®^, with NutriStem^®^ hESC XF supplemented with 5 μM ROCK inhibitor for 24 h (day -5). Cells were cultured in NutriStem^®^ hESC XF, changed daily. At high confluence (about 140%), after five days, the cells were treated with 12 μM Gsk3 inhibitor, CHIR99021 in RPMI medium supplemented with B27 without insulin for 24 h which was then changed to fresh RPMI/B27 without insulin. After 2 days, 5 μM of IWP2 was added for an additional two days and removed by changing the medium back to fresh RPMI/B27 without insulin for another two days. From day 7 cells were maintained in RPMI/B27 medium that was changed every 2 days.

### Gene expression analysis by qPCR

The cells were dissociated into a single-cell suspension using TrypLE™ for 2–4 min (for iPSCs) or collagenase type II (95 U/mL) (for iPSC-CMs) for 15 min and centrifuged for 5 min at 500 g and 4 °C. Total RNA was isolated by the PureLink^®^ RNA Mini kit (Rhenium); concentration and purity were measured using a Nano Drop 1000 spectrophotometer (Thermo Fisher Scientific). RNA from each sample was reverse transcribed into cDNA using the high capacity cDNA reverse transcription kit (Applied Biosystems, Foster City, CA). Gene expression analysis was performed using TaqMan gene expression assays (Applied Biosystems). Reactions were run on a StepOnePlus™ applied detection system (Applied Biosystems), and reaction conditions included 40 cycles of 95 °C for 10 s, followed by 60 °C for 30 s. Relative gene expression was calculated by the 2^–∆∆**Ct**^ method using GAPDH house-keeping gene. TaqMan assay data are detailed in Table 2.

**Table 2:** TaqMan assay data for quantitative RT-PCR

Standard gene abbreviation	Full gene name	Gene product	TaqMan gene expression assay ID
GAPDH	Glyceraldehyde-3-phosphate dehydrogenase	GAPDH	Hs99999905_m1
NKX2-5	NK2 homeobox 5	NKX2-5	Hs00231763_m1
ISL1	ISL LIM homeobox 1	ISL1	Hs00158126_m1
MYH6	Myosin, heavy chain 6, cardiac muscle, alpha	ɑMHC	Hs01101425_m1
MYH7B	Myosin, heavy chain 7B, cardiac muscle, beta	ßMHC	Hs00293096_m1
MYL2	Myosin, light chain 2, regulatory, cardiac, slow	MLC2v	Hs00166405_m1
MYL7	Myosin, light chain 7, regulatory	MLC2a	Hs00221909_m1
TNNT2	Troponin T type 2 (cardiac)	Troponin T	Hs00943911_m1
TNNI3	Troponin I type 3 (cardiac)	Troponin I	Hs00165957_m1
TNNC1	Troponin C type 1 (slow)	Troponin C	Hs00896999_g1
CACNA1C	Calcium channel, voltage-dependent, L type, alpha 1C subunit	Cav1.2	Hs00167681_m1
ATP2A2	ATPase, Ca^++^ transporting, cardiac muscle, slow twitch 2	SERCA2	Hs00544877_m1
CASQ2	Calsequestrin 2, Ca^++^ storage and transport, cardiac muscle	Calsequestrin 2	Hs00154286_m1

### Measurements of intracellular calcium transients and mechanical contraction

Intracellular calcium [Ca^2+^]i signals were measured with the HyperSwitch dual‐excitation and dual‐emission photometry system (IonOptix, MA, USA). Differentiated CMs were incubated for 10 min at 37 °C with medium containing 5‐μM Indo‐1AM (Molecular Probes) and Pluronic F‐127 (Life Technologies) at a dilution of 1:1, followed by a 10‐min wash with B27 supplement medium to remove excess dye. The cells were transferred to a perfusion chamber mounted on the stage of an inverted microscope and perfused with HEPES‐buffered Tyrode's solution (TB) containing (in mmole/L) NaCl 140, KCl 5.4, MgCl_2_ 1, sodium pyruvate 2, CaCl_2_ 1, HEPES 10, and glucose 10 (pH 7.4) warmed to 37 °C [27].

[Ca^+2^]i transients were measured by the F405/F480 ratio of Indo‐1AM fluorescence. Signals were recorded during stimulation at 0.25 Hz using a field stimulator (MyoPacer; IonOptix) through two platinum electrodes placed on the sides of the perfusion chamber. Data were analyzed using an IonWizard data acquisition system (IonOptix). Parameters were calculated from an average of 15–20 successive transients from 3 to 4 slides for each clone. [Ca^+2^]i transient amplitude was expressed as the delta amplitude from peak to baseline. Half width duration was measured from 50% raise to 50% decline of the [Ca^+2^]i transients.

Mechanical contraction was evaluated from 20 s movies using the MuscleMotion software [28]. Spontaneous beating CMs were continuously recorded using mvBlueFOX3 high-resolution (1024 × 1280) camera (Matrix Vision) at a capture rate of 120 frames/s. Half width duration was measured from 50% raise to 50% decline of the contraction transients. All data were collected from at least 6 different wells, 5–6 different areas in each well. All parameters were analyzed with one way ANOVA, multiple comparisons. p  < 0.05 was considered statistically significant.

### Autophagy measurements

For autophagy measurements, iPSCs and iPSC-CMs were treated according to CytoID kit (ENZ-51031) instructions using Rapamycin (final concentration 500 nM), Chloroquine (final concentration 20 μM) and DMSO (1:1000) for control. After 20 h of treatment with Rapamycin and Chloroquine together the medium was discarded, and the cells were detached using 1 mL of Versene (for iPSCs) and Collagenase II (for iPSCs-CMs) and picked up. The collected cells were washed with assay buffer X1 and centrifuged at 1100 rpm, 5 min. The supernatant was discarded; cells were frozen for future protein extraction by Western blot analysis. FACS analysis was performed immediately according to the CytoID kit instructions.

### Western blot analyses

Cells were lysed in RIPA lysis buffer (20 mM Tris–HCl, pH 7.5; 150 mM NaCl; 1% NP-40 or Triton -100; 0.5% sodium deoxycholate and 0.1% SDS) with 1 mM PMSF and a protease inhibitor (Sigma cat. No. S8830). Prior to separation, × 2 Laemmli sample buffer ]0.125 M Tris HCl, pH 6.8; 20% glycerol; 4% SDS; 0.004% bromophenol blue and 5% 2-mercaptoethanol (BME)] was added in a 1: 1 ratio. Subsequently, the samples were incubated for 5 min at 95 °C. 40 µg/lane of cell lysates was loaded on a 12% gel SDS-PAGE. The proteins were transferred to 0.2 m nitrocellulose membrane for 1 h at 100 V. The membrane was blocked using blocking buffer solution [(5% BSA in TBST (Tris-buffered saline, 0.1% Tween)] for 1 h at room temperature. The primary antibody, rabbit anti-LC3B (NB100-2220 Novus biologicals, USA, ~ 2 g/mL) was diluted in blocking buffer and incubated with the membrane overnight at 4 °C. The membrane was rinsed with TBST, 3 times for 5 min each. The blot was incubated with Working Solution [(mixing equal parts of the Stable Peroxide Solution and the Luminol/Enhancer Solution (Thermo Scientific™, 34080)] for 5 min. Signal intensities of LC3B bands were normalized against the internal control PonceauS [(Sigma P-3504) 0.1% PonceauS and 5% Acetic acid)], which label all proteins according to their concentrations. LC3B-II runs at 14–16 kDa while LC3B-I runs at 16–18 kDa.

### Statistical analysis

Statistical analysis was performed with GraphPad Prism version 6.05 for Windows (GraphPad Software, San Diego, CA). All variables are expressed as mean ± SEM. Results were analyzed either by one-way ANOVA with Tukey's post-hoc test for multiple comparisons or by two-tailed unpaired student's *t-*test. p  < 0.05 was considered statistically significant.

## Results

### Massive myocardial loss in a patient with mutated PLEKHM2

**Fig. 1:**
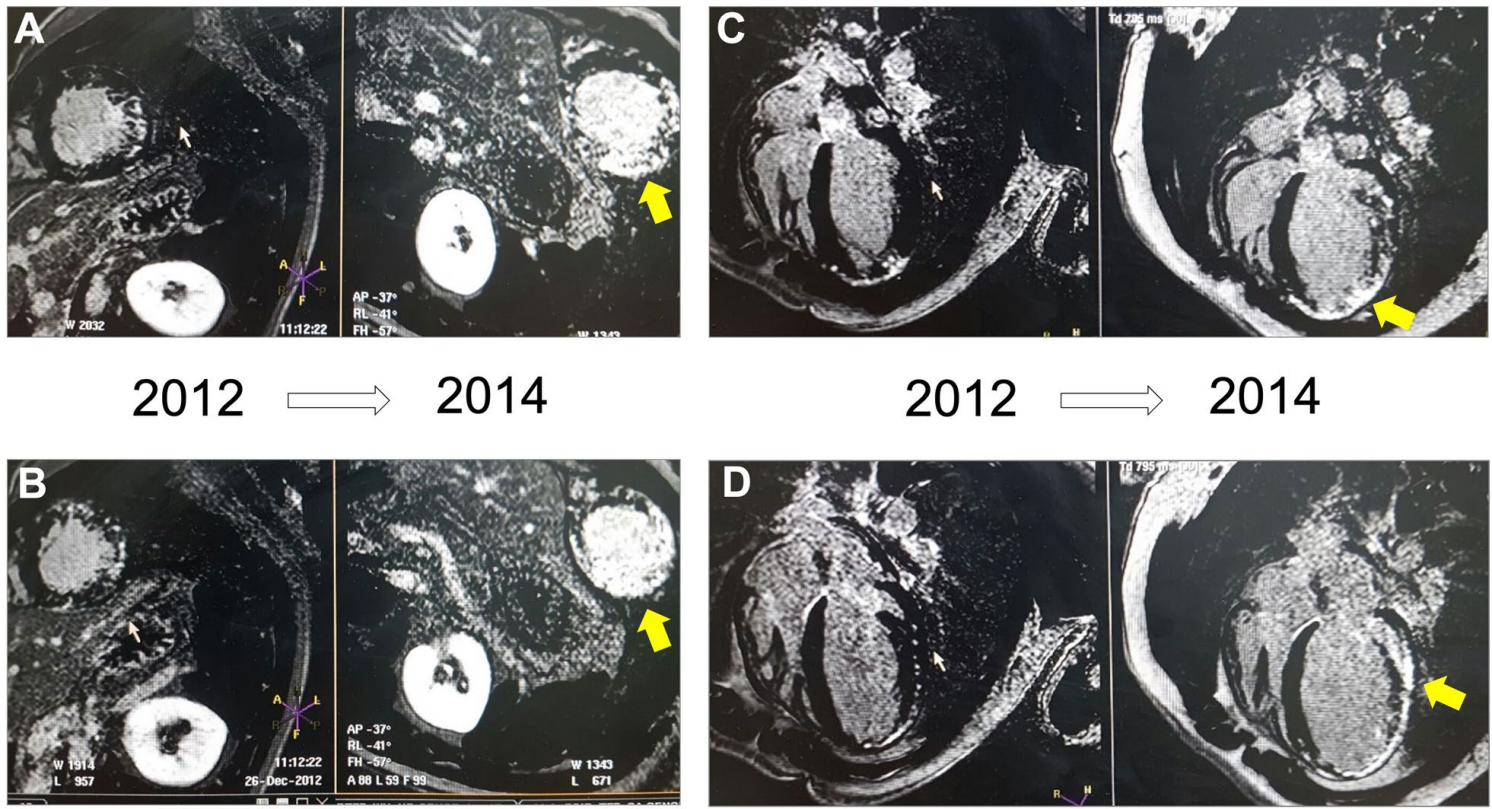
Massive myocardial loss in an adolescent patient with mutated PLKHM2 and familial cardiomyopathy. Sequential cardiac MRI studies; in all pictures left side shows an image from a 2012 exam and right side shows similar images from a 2014 exam. Late gadolinium enhancement imaging using phase-sensitive inversion recovery protocol (PSIR) is demonstrated. **A**, **B** short axis views, **C**, **D** four chamber views. Note focal patches of late gadolinium enhancement with prominent worsening in the second exams (yellow arrows). The patient was implanted with an ICD at the age 17.5 years, but had worsening heart failure and eventually died in hospital during preparations for cardiac transplantation. A skin biopsy obtained during ICD implantation was used to generate iPSCs that are examined in the present study (DCM-iPSCs, see text for details)

Clinical manifestations of four family members with mutated PLEKHM2 suffering from DCM and LVNC were previously reported [20]. As a follow up for this initial report, we now note that apart from cardiac dysfunction and dilation with LVNC features, accelerated cardiomyocyte death may be a prominent feature in these patients. Figure 1 demonstrates sequential cardiac MRI studies of one patient (II-2 in [20]), who participated in two consecutive studies in 2012 and 2014 at the age of 15 years and 17 years, respectively. While both figures demonstrate large transmural patches of fibrosis, the image at the later age demonstrates prominent worsening and progressive loss of myocardium that was viable in the younger age. In accordance, the patient had worsening heart failure symptoms and in 2016 died in hospital during preparations for cardiac transplantation. A connective tissue biopsy obtained during Implantable Cardioverter Defibrillator (ICD) implantation in 2014, was used to generate iPSCs that are the focus of the present study (DCM-iPSCs from patient O).

### Generation and characterization of patient specific iPSCs

**Fig. 2:**
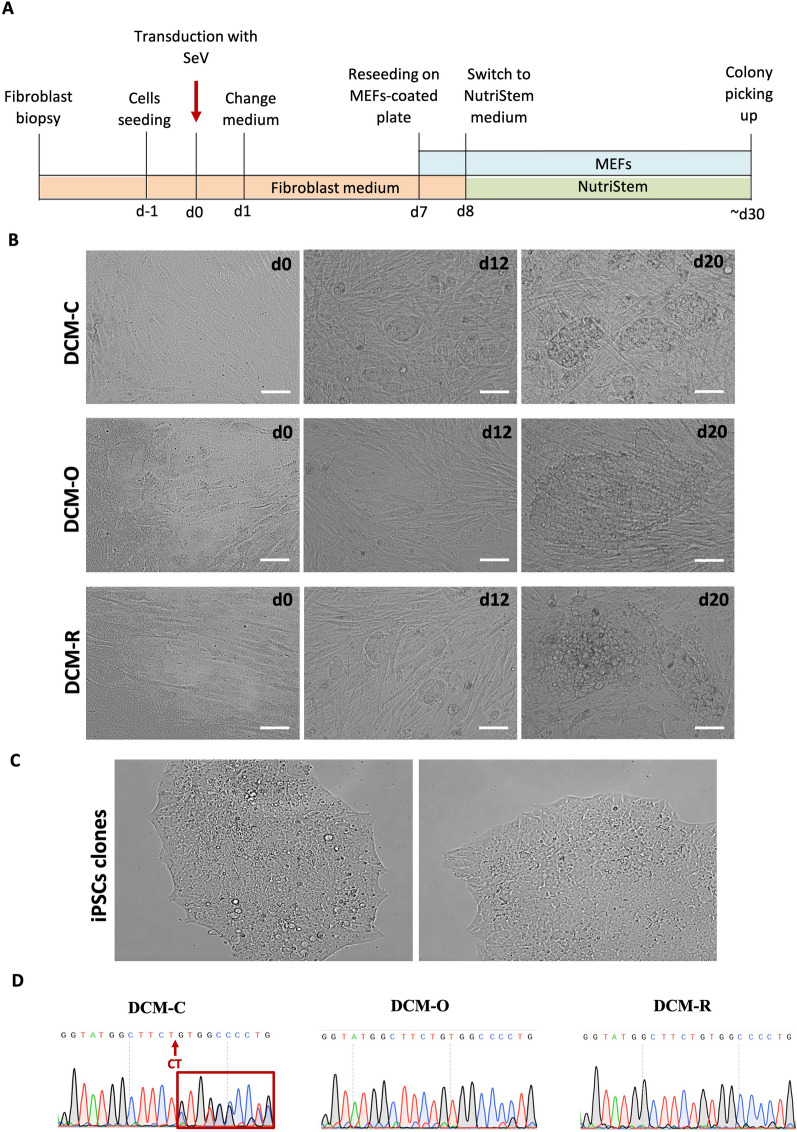
Generation of iPSCs using a chemically defined, integration-free methodology. **A** Time schedule of fibroblast reprogramming and iPSCs generation. **B** Phase contrast images of fibroblasts during reprogramming on different days and morphology of establishing DCM-iPS colonies. **C** Morphology of established iPS Lines. Cells were viewed using a FLoid^®^ Cell Imaging Station. **D** Sanger sequencing of the PCR product derived from the genomic DNA of the clones shows the complement sequence of mutation 2156_2157delAG in PLEKHM2 gene in DCM-iPSC lines. Scale bar 100 μm

To generate patient-specific iPSCs, primary fibroblasts were isolated from skin (DCM-C and DCM-R, healthy and patient, respectively) and internal connective tissue of another patient (DCM-O) (Table 3). These primary cells were expanded in vitro and reprogrammed with Sendai virus (SeV) expressing the Yamanaka 4 factors (Oct4, Sox2, Klf4, and c-MYC) (Fig. 2A and B).

**Table 3:** Clinical evaluation of patients and control (all from the same family)

	Patient age at onset and gender	Main clinical manifestations	Cells source
DCM-R (patient)	16, male	Severe LV dilatation. Severe global LV dysfunction. Ventricular tachycardia	Primary human fibroblasts from skin punch
DCM-O (patient)	13, male	Severe LV dilatation. Severe global LV dysfunction	Primary human fibroblasts from pacemaker replacement procedure
DCM-C (Control)	12, male	Control from the same family	Primary human fibroblasts from skin punch

Approximately 1 month after transduction, a distinct type of colonies that were flat and resembled hESC colonies were observed. hES cell-like colonies were picked and mechanically disaggregated into small clumps without enzymatic digestion (2–4 from each sample). These colonies were expanded and established as two individual iPSC lines for each patient (DCM-O1-iPSC, DCM-O7-iPSC, DCM-R4-iPSC and DCM-R7-iPSC) and healthy heterozygote sibling (control, DCM-C1-iPSC and DCM-C2-iPSC). Both patients’ and control derived iPSCs grew very well in culture and formed the characteristic pluripotent colonies (Fig. 2C). Sequencing the PCR product produced from the genomic DNA, we confirmed that DCM-O (DCM-O1-iPSC and DCM-O7-iPSC) and DCM-R (DCM-R4-iPSC and DCM-R7-iPSC) lines contain the specific 2156_2157delAG mutation in both alleles, while DCM-C (DCM-C1-iPSC and DCM-C2-iPSC) are heterozygous and contain a mutation only in one allele (Fig. 2D).

### iPSCs characterization

All generated iPSCs expressed high levels of embryonic stem-cell–associated antigens (SSEA4 and TRA-1-60) and OCT3/4 (Additional file 1: Fig. S1A–G), indicating successful reprogramming into pluripotent stem cells; the iPSC clones.

Karyotype analyses revealed that the cells were genetically stable with a normal complement of 46XY chromosomes (Additional file 1: Fig. S1H).

Sanger sequencing confirmed that DCM-O (DCM-O1-iPSCs and DCM-O7-iPSCs) and DCM-R (DCM-R4-iPSCs and DCM-R7-iPSCs) lines contained the specific 2156_2157delAG mutation in both alleles, whereas DCM-C (DCM-C1-iPSCs and DCM-C2-iPSCs) were heterozygous and contained mutations only in one allele (Additional file 1: Fig. S1I).

Upon spontaneous differentiation into embryoid-bodies (EBs), the iPSC clones showed lineage markers representative of the three embryonic germ layers, endoderm (AFP), mesoderm (SMA), and ectoderm (NF66) (Additional file 1: Fig. S1J–M), confirming their pluripotent nature.

Upon injection subcutaneously in the back of immune deficient NOD SCID mice, the iPSC clones formed teratomas with three germ layers (Additional file 1: Fig. S1N–P) [29].

### Monolayer differentiation to generate cardiomyocytes

Patient and control derived iPSCs were differentiated into cardiomyocytes (CMs) by using the small molecule monolayer differentiation protocol [25]. Two iPSCs clones for each patient and control (healthy heterozygote sibling) were used in subsequent experiments. All DCM-iPSC cultures were seeded at a density of 8*10^5^ cells/well, on Matrigel double-coated plate and treated with 12 μM CHIR99021 (on day 0) and 5 μM IWP2 (on day 3). Spontaneous contractions of iPSC-CMs were observed from day 8 of differentiation in all 6 lines.

### Patient-derived iPSCs-CMs show lower expression of cardiac specific genes

Quantitative Real-time PCR analysis of subsets of cardiac-associated genes representing different structural and functional activities in CMs, was performed on day 14 of differentiation; gene expression was normalized relative to GAPDH housekeeping gene and then compared to gene expression in differentiated DCM-C. The expression levels of most cardiac specific genes in patient-derived iPSC-CMs were considerably lower compared to the control iPSC-CMs.

**Fig. 3:**
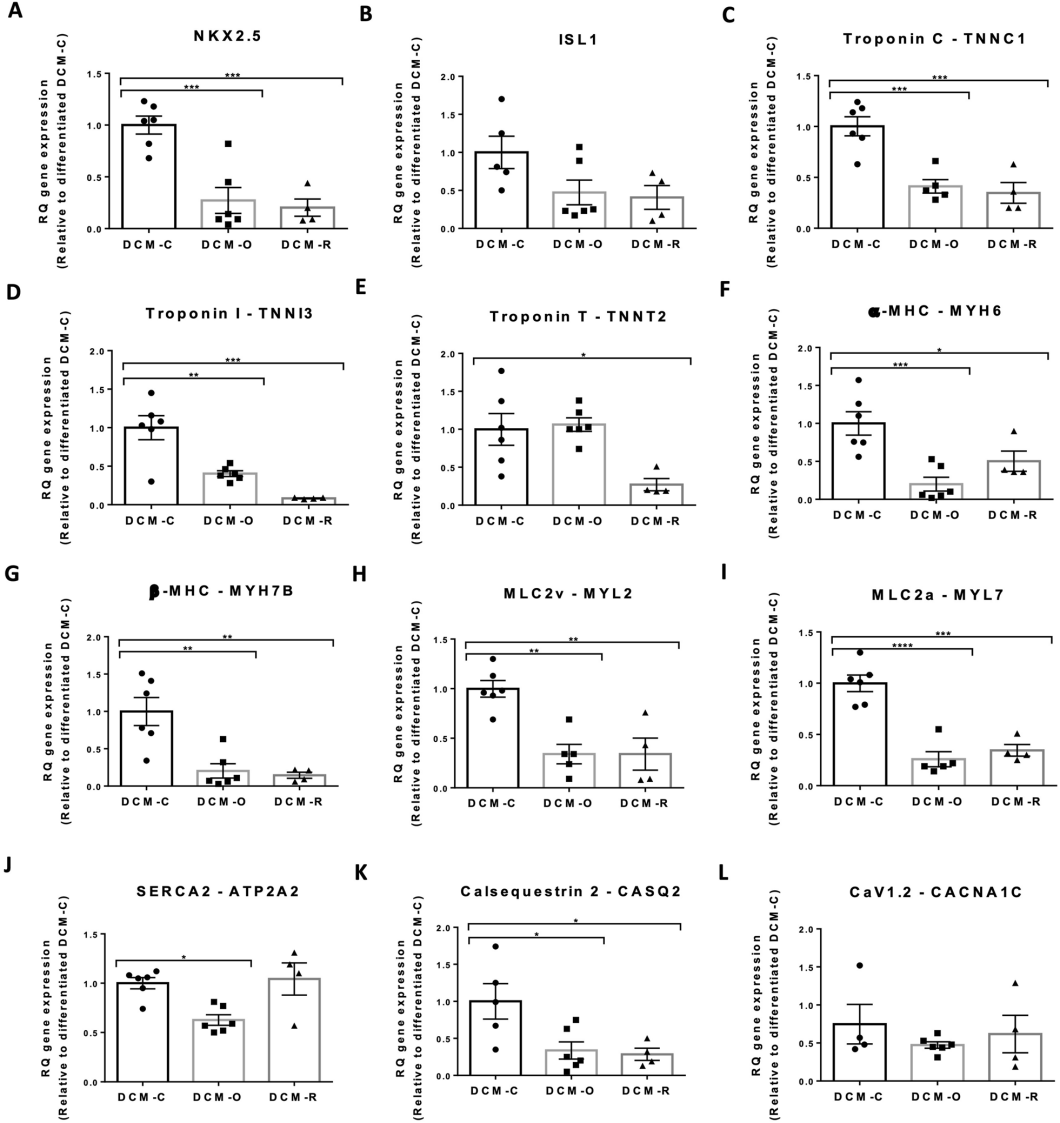
Gene expression of DCM-iPSCs-CMs (DCM-iPSCs-CMs clones C1 and C2, DCM-iPSCs-CMs clone O1 and DCM-iPSCs-CMs clone R4): qPCR readout of transcripts of cardiac progenitors NKX2.5 and ISL1 **A**,** B** contractile proteins, Troponin T, Troponin C and Troponin I **C**–**E** α-MHC and β-MHC, myosin heavy chain **F**, **G** and MLC2v and MLC2a, myosin light chains proteins that participate in electrical function **H**, **I**; CaV1.2, SERCA2 and Calsequestrin 2 **J**–**L**. Gene levels were normalized to GAPDH housekeeping gene and are represented relative to gene level expressed by non-differentiated DCM-C. n = 4–6 of biological replicates. Asterisks denote significant difference by 1-way ANOVA, *p < 0.05, **p < 0.01 ***, p < 0.001 and ****p < 0.0001

NKX2.5, an early marker of pre-cardiac mesoderm and cardiac progenitors, was significantly decreased in the patients compared to the control DCM-C- iPSCs-CMs (3.5 fold for DCM-O-iPSCs-CMs and fivefold for DCM-R-iPSCs-CMs, p < 0.001, Fig. 3A). The mesodermal and cardiac progenitor marker ISL1 showed a trend of decrease in expression in patient cells compared to control, although not reaching statistical significance (Fig. 3B).

The patient-derived iPSCs-CMs showed a considerable decrease in gene expression encoding for Troponin C (TNNC1), Troponin I (TNNI3) and Troponin T (TNNT2), which constitute a complex of structural proteins integral to heart contraction [30]. A 2.5-fold and a threefold decrease in TNNC1 expression in DCM-O-iPSCs-CMs and DCM-R-iPSCs-CMs, respectively, compared to control (p < 0.001 in both). A 2.5-fold and 12.5-fold decrease was noted in TNNI3 expression in DCM-O-iPSCs-CMs and DCM-R-iPSCs-CMs respectively compared to control (p < 0.01 and p < 0.001, respectively). A 3.5-fold decrease in TNNT2 expression in DCM-R-iPSCs-CMs (p < 0.05) was noted, while no difference was found in the levels of TNNT2 between DCM-O-iPSCs-CMs and DCM-C-iPSCs-CMs (Fig. 3C–E).

Additional contractile proteins, α and β -myosin heavy (MYH6 and MYH7B) chains and 2v and 2a-myosin light (MYL2 and MYL7) chains, exhibited decreased gene expression in the patient cells including: fivefold (DCM-O- iPSCs-CMs, p < 0.001), twofold (DCM-R- iPSCs-CMs, p < 0.05) for MYH6, fivefold (DCM-O- iPSCs-CMs, p < 0.01), 6.5 fold (DCM-R- iPSCs-CMs, p < 0.01) for MYH7B, threefold (DCM-O- iPSCs-CMs, p < 0.01), threefold (DCM-R- iPSCs-CMs, p < 0.01) for MYL2 and fourfold (DCM-O- iPSCs-CMs, p < 0.0001), threefold (DCM-R- iPSCs-CMs, p < 0.001) for MYL7 respectively (Fig. 3F–I).

The patient-derived iPSC-CMs exhibited a marked decrease in the expression of Calsequestrin 2, (CASQ2), a protein involved in the storage and transport of calcium ions [31, 32] (Fig. 3K); fivefold and sixfold decrease in DCM-O- iPSCs-CMs and DCM-R- iPSCs-CMs compared to control DCM-C- iPSCs-CMs (p < 0.05 in both). ATP2A2, encoding for the enzyme sarco(endo)plasmic reticulum calcium-ATPase 2 (SERCA2), responsible for Ca^2+^ pumping action showed decreased expression (1.5-fold, p < 0.05) in patient DCM-O-iPSCs-CMs compared to control DCM-C- iPSCs-CMs and no change in DCM-R- iPSCs-CMs (Fig. 3J). No differences were noted for CACNA1C, encoding for a protein constructing the α-subunit of the calcium channel CaV1.2 (Fig. 3L).

### FACS analysis

**Fig. 4:**
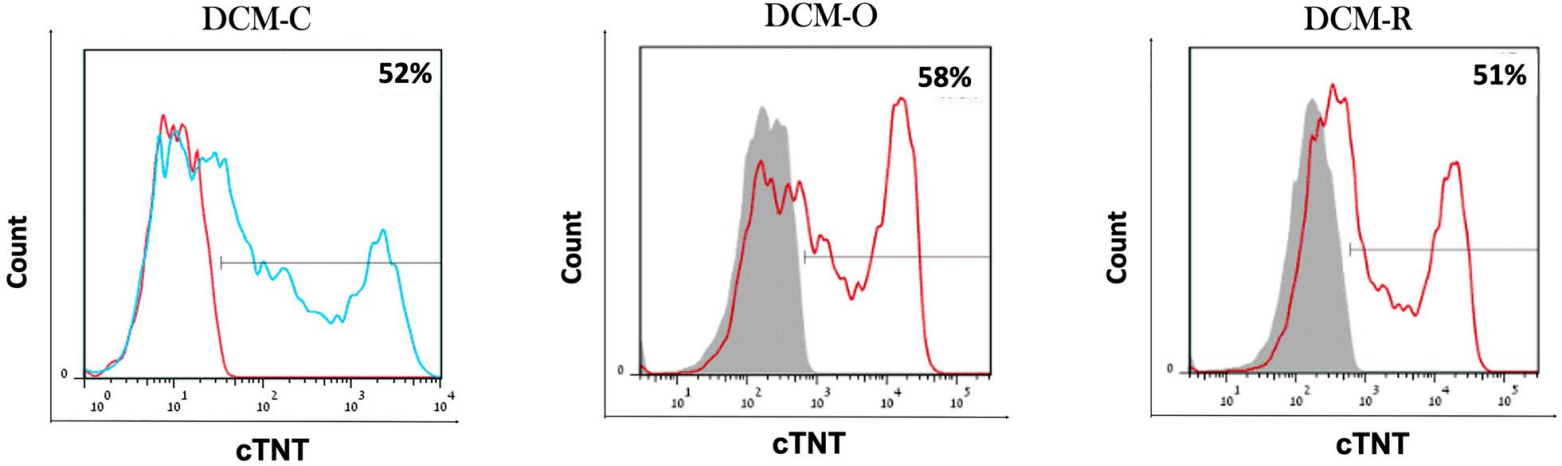
DCM-iPSCs differentiate into functional DCM-iPSC-CMs. The iPSC-CMs were cultivated on Matrigel-coated plates applying the small molecule monolayer cardiac differentiation protocol. An example of flow cytometry analysis of DCM-C-iPSC-CM, DCM-O-iPSC-CM and DCM-R-iPSC-CM for cTNT in 14-day old monolayer

By day 8–10 of differentiation, cardiac-related phenotypes were identified in all wells, such as spontaneous contraction. At day 14, the percentage of hESC-CMs was measured by staining the cells with cardiac Troponin T (cTNT) marker and analyzing by FACS. This analysis revealed that 41% (SE ± 10.4%), 49% (SE ± 12.7%), and 48.3 (SE ± 4.6%) of DCM-C-iPSC-CMs, DCM-O-iPSC-CMs, and DCM-R-iPSC-CMs, respectively, were cTnT-positive (Fig. 4).

### Patient-derived iPSCs-CMs exhibit less mature phenotype

**Fig. 5:**
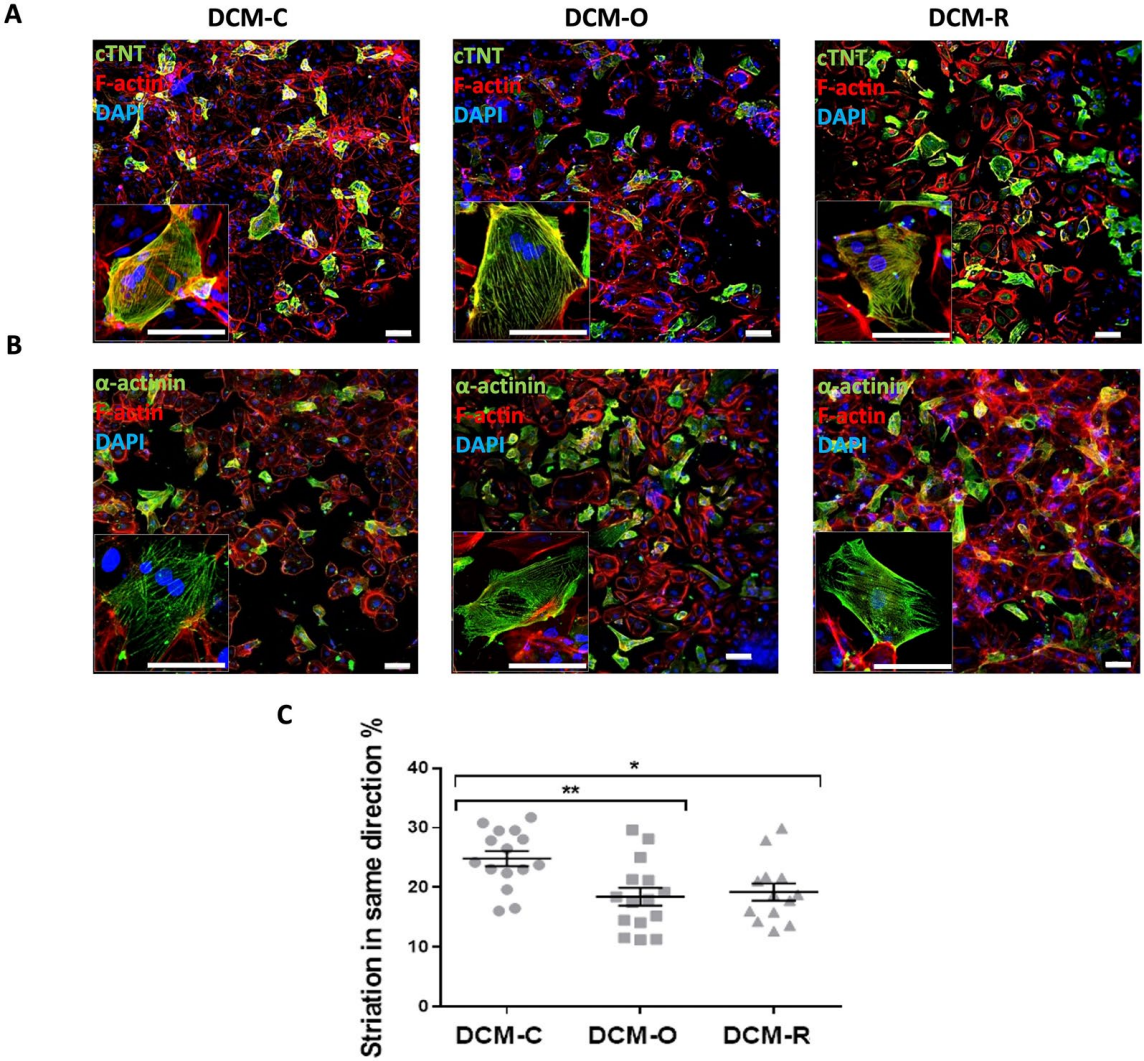
Distribution and morphology of DCM-iPSCs-CM. Immunostaining of cardiac troponin T (cTnT) and sarcomeric a-actinin at day 14 after differentiation. Single DCM iPSC-derived cardiomyocytes exhibited a cTNT (green) (**A**) or punctate sarcomeric a-actinin (**B)** double stained with F-actin (red) and DAPI (blue) distribution pattern. Enlarged views of the boxed areas show detailed a- actinin and cardiac troponin T staining patterns in the cells. Scale bars: 100 µm. Quantification of the extent of striation along the major axis of DCM-iPSCs-CMs stained with a- actinin in confocal images (n = 13–15) using Image J software (**C**). Asterisks denote significant difference by 1-way ANOVA, *p < 0.05, **p < 0.01

The presence of cardiac specific proteins and their spatial organization within the DCM-iPSC-CMs were determined by immunostaining with either anti-cardiac troponin T (cTnT) or sarcomeric α-actinin, two markers specific to CMs, coupled with immunostaining of F-actin. Positive double staining distinguished the CMs from non-myocytes, the latter were stained positive only for F-actin. Both control and patient-derived DCM-iPSCs-CMs expressed the sarcomeric proteins cTnT (Fig. 5A) and sarcomeric α-actinin (Fig. 5B) at day 14 post differentiation. cTnT appeared as longitudinal, punctuate, fibrous striations and the sarcomeric α-actinin participated in highly organized cytoskeletal structure. The cTnT and sarcomeric α-actinin positive cells were arranged in beating foci, surrounded by non-myocytes.

The phenotypic maturity of the stem cell-derived CMs was scored based on the degree of sarcomeric structural organization [33]. The extent of striation along the major axis of iPSC-CMs stained with α-actinin (Fig. 5C) was quantified using Image J software and the Orientation J Distribution plugin [34]. OrientationJ calculates the distribution of fiber orientations in an image by evaluating the structure tensor. Briefly, the immunostained iPSCs derived CMs projections were transformed into binary images, and the transformed image was analyzed using OrientationJ for cell directionality. The results of this analysis showed that most iPSC-CMs in the 14-day differentiated cultures presented an immature sarcomere fiber morphology, yet it is possible to identify that both patient cells had fewer CMs containing organized sarcomere structure compared to control healthy cells (Fig. 5C).

### Mutation in PLEKHM2 alters calcium transients and mechanical contraction in patient iPSC-CMs

**Fig. 6:**
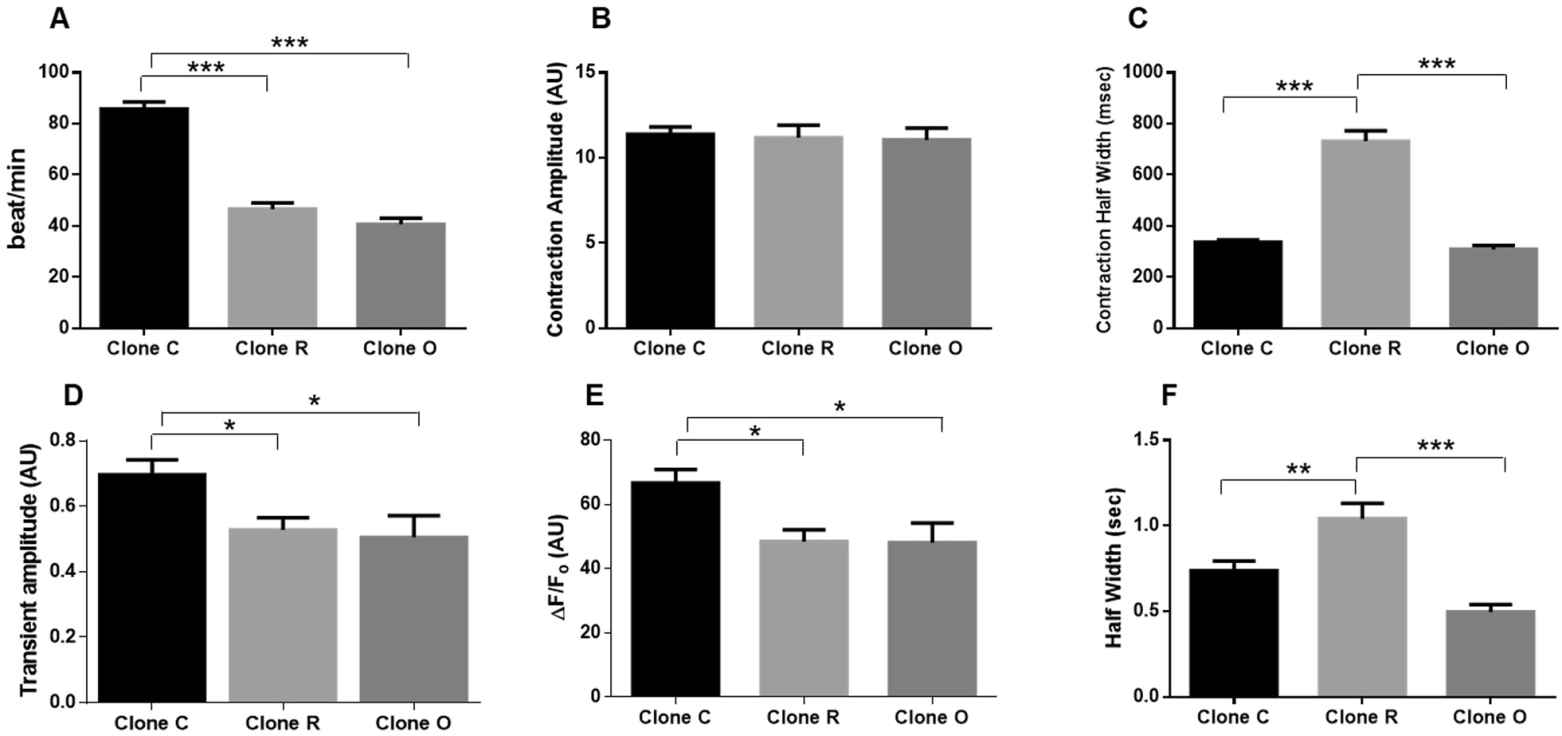
Calcium transients and mechanical contraction were altered in PLEKHM2 mutated DCM-iPSC-CMs. **A** Spontaneous beat rate/min (**A**) and Mechanical contraction (**B**, **C**) were measured using MuscleMotion software. Two clones were analyzed from each patient (DCM-iPSCs-CMs clones R4 and R7, n = 25 and DCM-iPSCs-CMs clones O1 and O7, n = 40) or control (DCM-iPSCs-CM clones C1 and C2, n = 77). **B** Contraction amplitude represents an average of the maximum peak of contraction during spontaneous beatings. **C** Half width represents the time duration of 50% raise to 50% decline of the contraction-relaxation signal. Intracellular calcium transients (**D**–**F**) were measured with the HyperSwitch dual‐excitation and dual‐emission photometry system (IonOptix, MA, USA) during stimulation at 0.25 Hz. **D** An average of [Ca^2+^]i transients amplitude. **E** ∆F/F0 represents the % of [Ca^2+^]i change during contraction relaxation cycle. **F** Half width represents the time duration of 50% raise to 50% decline of the [Ca^+2^]i transients (only one clone was measured; Clone C2, n = 17, Clone R4 n = 17 and Clone O7 n = 8). All statistics were done using One way ANOVA with multiple comparisons and present as average ± SE *p < 0.05, ***p < 0.001

We examined mechanical contraction and intracellular [Ca^2+^]i transients of homozygously mutated (Clones R and O) and control (Clone C) iPSCs-CMs grown for 15–19 days. All samples showed spontaneous beating responses and could also be captured by external pacing at 0.25 Hz. Figure 6A shows the average spontaneous beating rate of the different clones. While the healthy clone had a beating rate of ~ 85 beat/min, slower beating rates were noted in both PLEKHM2-mutated clones (Clone R, ~ 46 beat/min and Clone O, ~ 40 beat/min, p < 0.001). Analysis of peak contraction amplitude was not different between the clones **(**Fig. 6B**)**. However, the kinetics of contraction demonstrate twofold slower (p < 0.001) contraction-relaxation cycle in iPSC-CMs from clone R as compared to healthy iPSCs-CMs (Clone C). IPSCs-CMs from clone O did not show significant changes in contraction-relaxation measurements compared to the control clone **(**Fig. 6C**)**. Due to the above noted changes in beating rate and in order to evaluate calcium handling under standard conditions, the [Ca^2+^]i measurements were performed under constant pacing at a uniform frequency. We used pacing at 0.25 Hz since the mutated iPSCs-CMs did not capture at higher pacing rates. [Ca^2+^]i transients signals were measured in three clones (C2, R4 and O7). Figure 6D–F indicates that both mutated clones (R4 and O7) demonstrate a decrease in [Ca^2+^]i transient amplitude **(**Fig. 6D, p < 0.05**)** and ∆F/F0 values **(**Fig. 6E, p < 0.05**)** as compared to the control clone C2. In addition, as also demonstrated in the contraction analysis kinetics **(**Fig. 6C**)**, there was a different tendency between the mutated clones with slower [Ca^2+^]i kinetics for clone R4 (p < 0.01) but no changes in [Ca^2+^]i kinetics for clone O7 as compared to the control (clone C2) **(**Fig. 6F**)**.

### Comparison of PLEKHM1 and PLEKHM2 gene expression

**Fig. 7:**
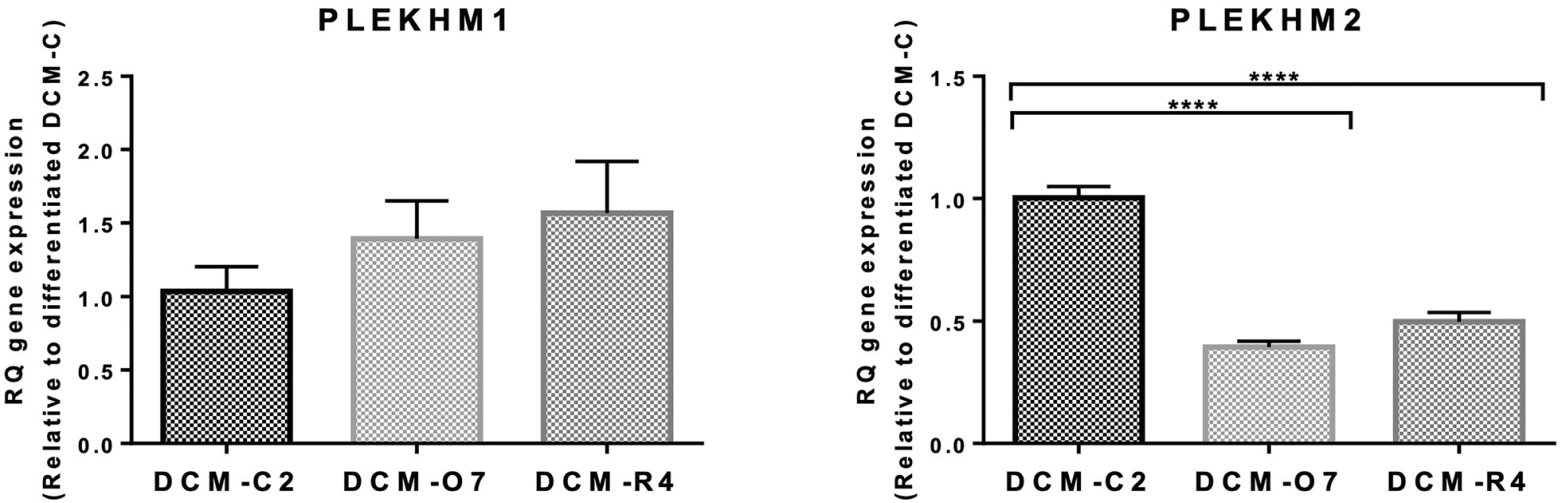
Gene expression of *PLEKHM1* and *PLEKHM2* in mutated area of DCM-iPSCs and DCM-iPSCs-CMs: qPCR readout of transcripts of PLEKHM1 and PLEKHM12. Gene levels were normalized to GAPDH housekeeping gene and are represented relative to gene level expressed by non-differentiated DCM-C. n = 4–6 of biological replicates. Asterisks denote significant difference by 1-way ANOVA, *p < 0.05, **p < 0.01 ***, p < 0.001 and ****p < 0.0001

*PLEKHM1* has a role in regulation of autophagosome–lysosome fusion (similar to PLEKHM2) during autophagy [35, 36] and may compensate for the reduced activity rate of mutated PLEKHM2. Therefore, we assessed the expression of PLEKHM2 and PLEKHM1 using patient’s and control RNAs. Our findings indicate that there is almost twofold decrease in the RNA level of PLEKHM2 in patient’s differentiated iPSC-CMs clones as compared to the control. There was no change in the expression level of PLEKHM1 mRNA between patients and control, suggesting that PLEKHM1 does not compensate for the mutated PLEKHM2 (Fig. 7).

### PLEKHM2-mutated DCM iPSC-CMs exhibit impaired autophagy

We evaluated the autophagy in DCM-C-iPSCs-CMs, DCM-O-iPSCs-CMs and DCM-R-iPSCs-CMs following treatment with rapamycin (RP; a known inducer of autophagy) and chloroquine (CQ; CQ known as lysosome inhibitor and also acting as an autophagy inhibitor by impairing autophagosome fusion with lysosomes [36]). The autophagic activities were compared to the basal autophagy activity levels following treatment with DMSO used as the solvent for RP and CQ.

**Fig. 8:**
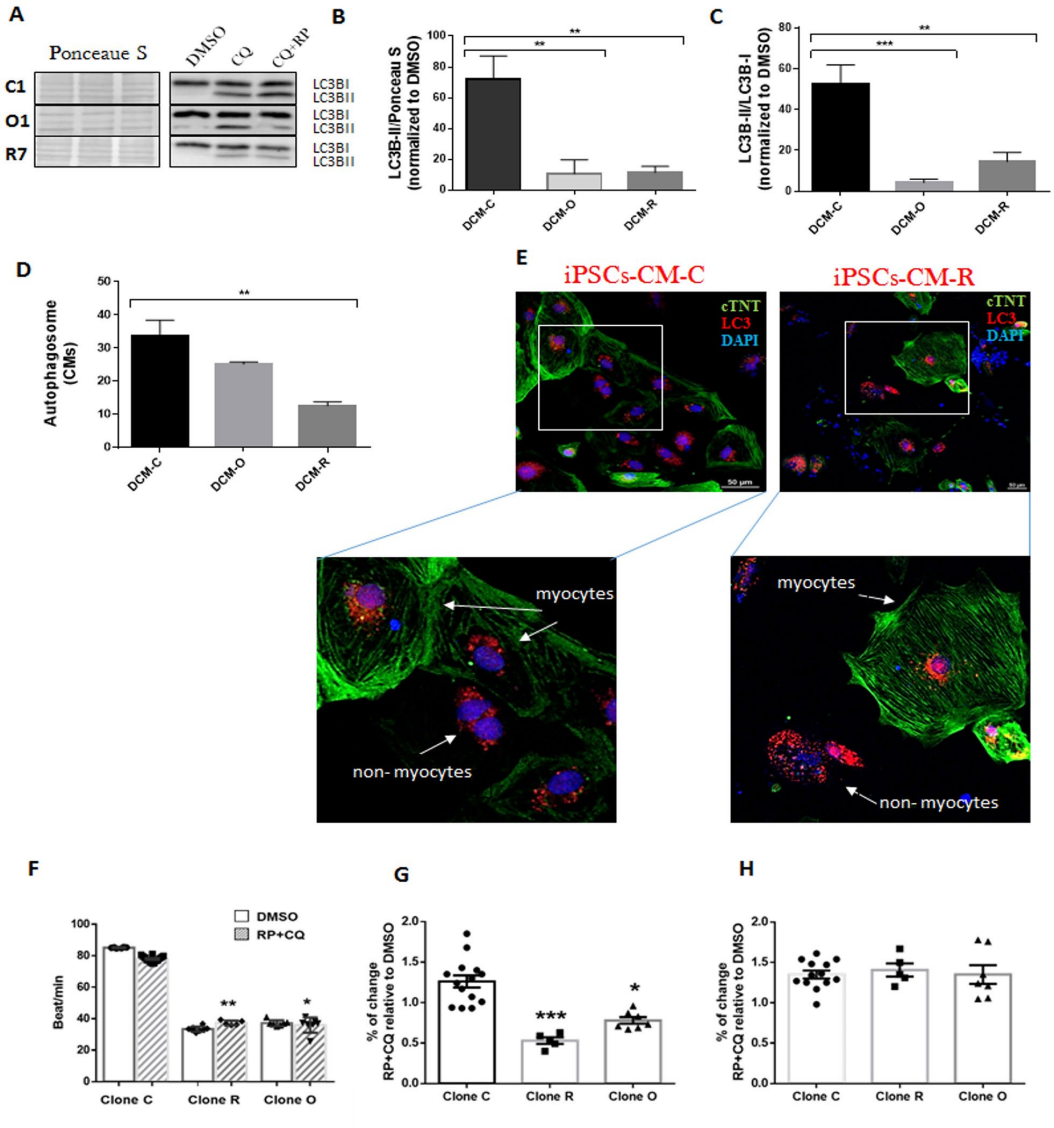
Impairment autophagy and physiology response in PLEKHM2 mutated DCM-iPSCs-CMs. Cells were treated with 0.1% DMSO or 20 μM Cloroquine (CQ) and 500 nM Rapamycin (RP) for 20 h. RP + CQ treated samples were related to DMSO treatment for each cell line to compare the autophagic response independent of varying basal expression levels among cell lines. Western blot analysis with representative immunoblots (performed on all clones) (**A**)**,** quantification of LC3B-II normalized to Ponceau S as a loading control (performed on all clones, n = 2–3 of biological replicates) (**B**), quantification of LC3B-II/ LC3B-I ratio (performed on all clones, n = 2–3 of biological replicates) (**C**). FACS analysis using CytoID kit (**D**). Immunostaining of DCM-iPSCs-CM-C and DCM-iPSCs-CM-R (**E**) Immunostaining of cardiac troponin T (cTnT) and LC3B at day 28 of differentiation after treatment with Rapamycin (500 nM) and Chloroquine (20 μM). Single DCM iPSC – derived cardiomyocytes exhibited a cTNT (green) double stained with LC3B (red) and DAPI (blue) distribution pattern. Scale bars, 50 µm. Spontaneous beat/min rate (**F**) and mechanical contraction (**G**, **H**) were measured following treatment with DMSO or RP and CQ. One clone from each patient was analyzed using MuscleMotion software (DCM-iPSCs-CM clones C1, DCM-iPSCs-CMs clone R7 and DCM-iPSCs-CMs O1) and the results (**G**, **H**) are represented as the percentage of the change accrued following RP + CQ treatment. Asterisks represent significance in compared to clone C. **G** Contraction amplitude represents an average of the maximum peak of contraction during spontaneous beatings. **H** Half width represents the time duration of 50% raise to 50% decline of the contraction-relaxation signal. Intracellular calcium transients. Asterisks denote significant difference by 1-way ANOVA, *p < 0.05, **p < 0.01 ***p < 0.001 and ****p < 0.0001

In the process of autophagy, microtubule-associated protein 1 light chain 3-I (LC3BI) is converted into microtubule-associated protein 1 light chain 3-II (LC3BII) that is incorporated into the surface of the phagophore [37]. Therefore, the increase in the expression ratio of LC3B-II/LC3B-I is one of the markers for identifying the presence of autophagosomes. Autophagic activity was assessed following treatment with RP and CQ and the accumulation of LC3B-II as well as the LC3B-II / LC3B-I ratio were measured by Western blotting using specific antibodies (Fig. 8A–C). As seen, protein levels of LC3B-II were 7- and 6.5-fold lower for DCM-O-iPSCs-CMs and DCM-R-iPSCs-CMs, respectively, compared to control healthy DCM-C-iPSC-CMs (p < 0.01, Fig. 8A, B**)**. The LC3B-II/LC3B-I ratio was 4- and 13-fold lower for DCM-O-iPSC-CMs (P < 0.01) and DCM-R-iPSC-CMs (p < 0.001), respectively, compared to the control DCM-C-iPSC-CMs (Fig. 8A–C).

In addition, the CYTO-ID^®^ Autophagy Detection Kit was used to detect autophagy activity according to the amount of accumulated autophagosomes within the cells [38]. The levels of autophagosomes in the cells were quantified in response to RP and CQ and were normalized to the number of autophagosomes at the basal level. After autophagy manipulation, the amount of autophagosomes in patient’s DCM-R-iPSCs-CMs was significantly lower (p < 0.05) and similar tendency was shown in patient DCM-O-iPSCs-CMs compared with control DCM-C- iPSCs-CMs (Fig. 8D).

Collectively, the decreased level of LC3B-II according to Western blotting assay and the lesser amount of phagophores and autophagosomes in both patients' iPSC-CMs according to flow cytometry analysis indicate that the mutation in PLEKHM2 reduces autophagy activity in both cardiomyocytes and non-myocytes, which consist of about half the cells in the tissue cultures. Immunostained pictures show that LC3B puncta, representing phagosomes, are produced by both cardiomyocytes and non-myocytes in patient and control cultures (Fig. 8E).

Having validation for autophagy differences between patient and control iPSC-derived CMs, we then proceeded to examine whether and to what extent this has an effect on CM contractility. We examined CM contractility in response to treatment with DMSO or following autophagy manipulation with RP and CQ. The mutated cells beat significantly slower at baseline (DMSO treatment, p < 0.01) in comparison to the control cells as was demonstrated before **(**Fig. 6A**)**. Treatment with RP and CQ did not change the beat/min rate in any clone (Fig. 8F), suggesting that autophagy may not be the direct cause for the slower beating that has been demonstrated in mutant cells. However, contraction amplitude analysis shows a significant negative inotropic effect in both mutated clones following treatment with RP and CQ (Fig. 8G, p < 0.006). This decrease in contraction amplitude was not accompanied by a change in the kinetics of contraction **(**Fig. 8H**)** in comparison with the control CMs.

## Discussion

The clinical manifestations of the family members with homozygously mutated PLEKHM2 were reported previously [20]. In a follow-up study reported here, sequential cardiac MRI analyses of one patient (DCM-O) identified an important additional feature, a rapid transmural loss of myocardial tissue that is replaced by patchy scar formation. Interstitial and perivascular fibrosis is a hallmark feature of systolic heart failure in general [39–42]. However, the accumulating amount of fibrosis observed in the DCM-O patient is not a typical finding in other forms of DCM. This newly identified feature of the mutated PLEKHM2-dependent cardiomyopathy indicates that the function of PLEKHM2 protein is crucial to maintain myocardial tissue viability over time. To further verify the function of PLEKHM2 in heart and how the mutation identified in the patients leads to their clinical manifestations, we established iPSC-derived cardiomyocytes (iPSC-CMs) from two of the patients and their healthy brother.

Human iPSC-CMs have been shown to faithfully model the disease phenotype associated with single gene mutations known to affect CM function [43–45] (reviewed in [5]). Among the recent examples: iPSC model of cardio-facio-cutaneous syndrome (CFCS) resulting from germline mutations in BRAF, enabled to show that differentiation of mutated iPSCs towards CMs leads to fibroblast-like cells that influence CM hypertrophy through TGFβ [46]. The causality of a missense mutation in SPEG, which was identified in patients with DCM, was demonstrated by functional studies in iPSC-CMs harboring the mutation by CRISPR/Cas9-mediated genome editing. The iPSC-CMs showed aberrant calcium homeostasis, impaired contractility, and sarcomeric disorganization, recapitulating the hallmarks of DCM [47]. Danon disease, a familial cardiomyopathy associated with impaired autophagy, was modeled using human iPSC-CMs from patients with mutations in the gene encoding lysosomal-associated membrane protein type 2 (LAMP-2) [48].

In the present study, two independent patient samples and one healthy control were reprogrammed and two clones from each patient were studied. Upon differentiation, mixed cell populations of CMs and non-myocytes were generated. Such mixed populations of cells were also shown for other hiPSC models for cardiac disease [49].

After 14 days of differentiation, the viability of patients’ and control iPSC-CMs was similar. However, the patients’ iPSC-CMs presented delayed maturation status in comparison to control iPSC-CMs, as demonstrated by the lower expression level of several cardiac-related genes, such as NKX2.5, MHC, MLC, Troponins and CASQ2 in patient cells. Furthermore, despite the fact that the protein level of cardiac troponin T was similar in the control and mutant—DCM-O iPSC-CMs after differentiation, the sarcomeres of control iPSCs-CM cells became more oriented and aligned. Sarcomeric alignment and organization are critical for electromechanical coupling and generation of a contractile force, and provide an indication for hESC-CM maturation [50]. Better sarcomere alignment and the significantly higher mRNA level of Troponin I (TNNI3), which is considered to be an adult isoform [30, 51, 52], as well as expression of the ventricular marker MLC2v [52], point to a better progression of the control iPSC-CMs towards maturation in comparison to the PLEKHM2-mutated cells. Finally, patients’ iPSC-CMs presented a decline in the beating rate in comparison to the control iPSC-CMs.

The negative chronotropic effect and delayed maturation of iPSCs-CM demonstrated in the PLEKHM2-mutated cells led us to further examine the mechanical contraction and intracellular calcium [Ca^2+^]_i_ signaling. Both the contractile force and beating rate of CMs are dependent on the amplitude and kinetic properties of Ca^2+^ transients. We demonstrate a mild decrease in [Ca^2+^]_i_ amplitude as well as a decrease in [Ca^2+^]_i_ magnitude (ΔF/F0) that occurred during contraction-relaxation cycle in the mutated cells. However, this effect was not observed when mechanical contraction amplitude was measured. Calculating the contraction and calcium kinetics showed that only clone R had a longer contraction-relaxation cycle time (as indicated by the half width time) compared to control.

Altered Ca^2+^ cycling and cardiac contraction is well known in heart failure [53] and was also demonstrated in iPSC-CMs with mutations causing defective sarcomere structures: S635A- in RBM20 [54], E1680K in SPEG [47] R173W in TNNT2 [55]. Recently hiPSC-CMs generated from patients with delayed-onset LVNC linked to a missense mutation, the G296S, in GATA binding protein 4 (GATA4) showed impairments in contractility, calcium handling, and metabolic activity [56]. Thus, the abnormalities we observed may not be caused by the direct effect of mal-function of PLEKHM2 but by the secondary effect of the reduced maturation to cardiomyocytes and defective sarcomeric structure of the patients’ iPSC-CMs.

The mutated PLEKHM2 eliminates the PH domain, which is required for generating the autophagolysosome and our previous study demonstrated a defect in autophagy flux in patients’ skin fibroblasts [20]. We first ascertained that the mRNA level of PLEKHM2 in patients iPSC-CMs is lower as compared to its expression in control iPSC-CMs and that there is no compensation of the PLEKHM2 activity by PLEKHM1 which functions similarly to PLEKHM2 [35, 36, 57–59], by demonstrating that the mRNA level of PLEKHM1 was similar in patient and control iPSC-CMs. Indeed, we found significantly lower levels of LC3BII in patients’ cultures of iPSC-CMs which consist of both cardiomyocytes and non-myocytes and lower levels of LC3BII-positive autophagosomes that were demonstrated in patients’ iPSC-CMs. Although we didn’t directly measure the reduction in autophagosomes in non-myocytes in the culture we think that they too have less autophagosomes as we demonstrated in the patient’s fibroblasts and in our unpublished studies in a mouse model. Another model of iPSC-CMs defective in autophagy was produced from two Danon disease patients with different mutations in lysosomal-associated membrane protein type 2 (LAMP-2) [48]. These iPSC-CMs demonstrated impaired autophagy, increased cell size, increased expression of natriuretic peptides, and abnormal calcium handling, compared to control iPSC-CMs. as well as excessive amounts of mitochondrial oxidative stress and apoptosis. However, in contrast to our findings their iPSC-CMs presented high cardiac differentiation efficiency, similarly to the control iPSC-CMs. These cells presented earlier autophagic vesicles that were also supported by higher LC3B II levels and reduction in the late autophagic vesicles. Danon iPSC-CMs from all lines were significantly larger than WT iPSC-CMs, phenocopying the hypertrophy observed in Danon patients and longer calcium decay times compared to control. Additionally, Danon iPSC-CMs demonstrated excessive amounts of mitochondrial oxidative stress and apoptosis.

Autophagy contributes to the maintenance of normal cardiovascular function and thus functions as a protective mechanism under physiological conditions [60]. However, autophagic response can cause cell death by defective autophagosome clearance [61]. The lower levels of autophagic activity that were demonstrated in patients’ iPSC-CM as compared to control iPSC-CM may affect the removal of damaged organelles and therefore promote heart failure and early mortality. Indeed, mice models with cardiac-specific autophagy-related gene deficiency have shown that autophagy disorders play a crucial role in disrupting cardiac structure and function, leading to cardiac dysfunction over time [62, 63].

## Conclusion

We developed a novel disease in a dish model from *PLEKHM2*-mutated patient cells to examine the mechanism that leads to this disease phenotype. This in vitro patient-specific cellular model is the closest possible model of the human heart and will be useful to study the underlying molecular mechanisms and test new therapeutic strategies. We suggest that the basic impairment in autophagy which affects the differentiation into mature cardiomyocytes in the model affects the function of all cell types in the patients’ hearts. The structural defects caused in the cardiomyocytes affect the Ca^2+^ handling and the contraction of the heart and the defective autophagy renders reduced capability to respond to different effects of stress during life time. This model can contribute to the study of the molecular mechanisms that cause impaired autophagy and further on could lead to development of effective treatments for cardiac dysfunction and relief syndromes in these patients [64].

## Supplementary Information

Figure S1: iPSC characterization. Quantitative flow cytometry and immunofluorescence analysis were applied using anti NANOG (A, D, E), Oct ¾ (B, D, F) and Sox2 (C, D, G) antibodies for all iPSC lines. Representative images of karyotype analysis in DCM cell lines (H). DNA sequencing shows the complement sequence of mutation 2156_2157delAG in PLEKHM2 gene in DCM-iPSC lines (I). Spontaneous differentiation steps by EB formation using the hanging drop method (J). All EBs were stained by anti-SMA, anti-NF68, and ant- AFP antibodies (K-M). In vivo teratoma assay of the DCM-iPSCs. Scale bar, 100 μm (N-P). **Additional file 1.**

## Data Availability

All data generated or analyzed during this study are included in this published article.

## Abbreviations

AFP: Alpha fetoprotein
APC: Allophycocyanin
Arl8: ADP-ribosylation factor-like protein 8
ATP: Adenosine triphosphate
BSA: Bovine serum albumin
CM: Cardiomyocyte
CQ: Chloroquine
cTNT: Cardiac troponin T
DAPI: 4′,6-Diamidino-2-phenylindole
DCM: Dilated cardiomyopathy
DMEM: Dulbecco's modified eagle medium
DMSO: Dimethyl sulfoxide
EBs: Embryoid bodies
FACS: Fluorescence activated cell sorter
FBS: Fetal bovine serum
GAPDH: Glyceraldehyde 3-phosphate dehydrogenase
GSK3: Glycogen synthase kinase 3
hESC: Human embryonic stem cells
hESC-CM: Human embryonic stem cell-derived cardiomyocytes
hiPSC: Human induced pluripotent stem cells
hiPSC-CM: Human induced pluripotent stem cells-derived cardiomyocytes
IWP2: Inhibitor of Wnt production-2
KLF4: Kruppel like factor 4
LAMP2: Lysosomal associated membrane protein 2
LC3: Microtubule-associated protein 1A/1B-light chain 3
LC3BI: Microtubule-associated protein 1-light chain 3-I
LC3BII: Microtubule-associated protein 1-light chain 3-II
LSCM: Laser scanning confocal microscope
MEFs: Mouse embryonic fibroblasts
MEM-NEAA: Minimum essentiall medium non-essential amino acid solution
MRI: Magnetic resonance imaging
NF66: Neurofilament alpha-internexin
NOD SCID: Nonobese Diabetic—severe combined immunodeficiency
OCT: Octamer-binding transcription factor
PBS: Phosphate buffered saline
PLEKHM1: Pleckstrin homology domain-containing family M member 1
PLEKHM2: Pleckstrin homology domain-containing family M member 2
PMSF: Phenylmethylsulfonyl fluoride
qPCR: Quantitative PCR
RIPA: Radio immunoprecipitation assay
ROCK: Rho-associated protein Kinase
RP: Rapamycin
RPMI: Roswell park memorial institute
SDS: Sodium dodecyl sulfate
SeV: Sendai virus
SKIP: SifA and kinesin interacting protein
SMA: Smooth muscle actin
SOX2: SRY-box transcription factor 2
SSEA: Stage-specific embryonic antigen
TBST: Tris-buffered saline, 0.1% Tween
TRA: Tumor rejection aantigens
