# Supplementary figures and images for: Functional defects in hiPSCs-derived cardiomyocytes from patients with a PLEKHM2-mutation associated with dilated cardiomyopathy and left ventricular non-compaction
Source: Biol Res. 2023 Jun 23;56:34. doi: 10.1186/s40659-023-00442-5 (PMC10288792; doi:10.1186/s40659-023-00442-5)

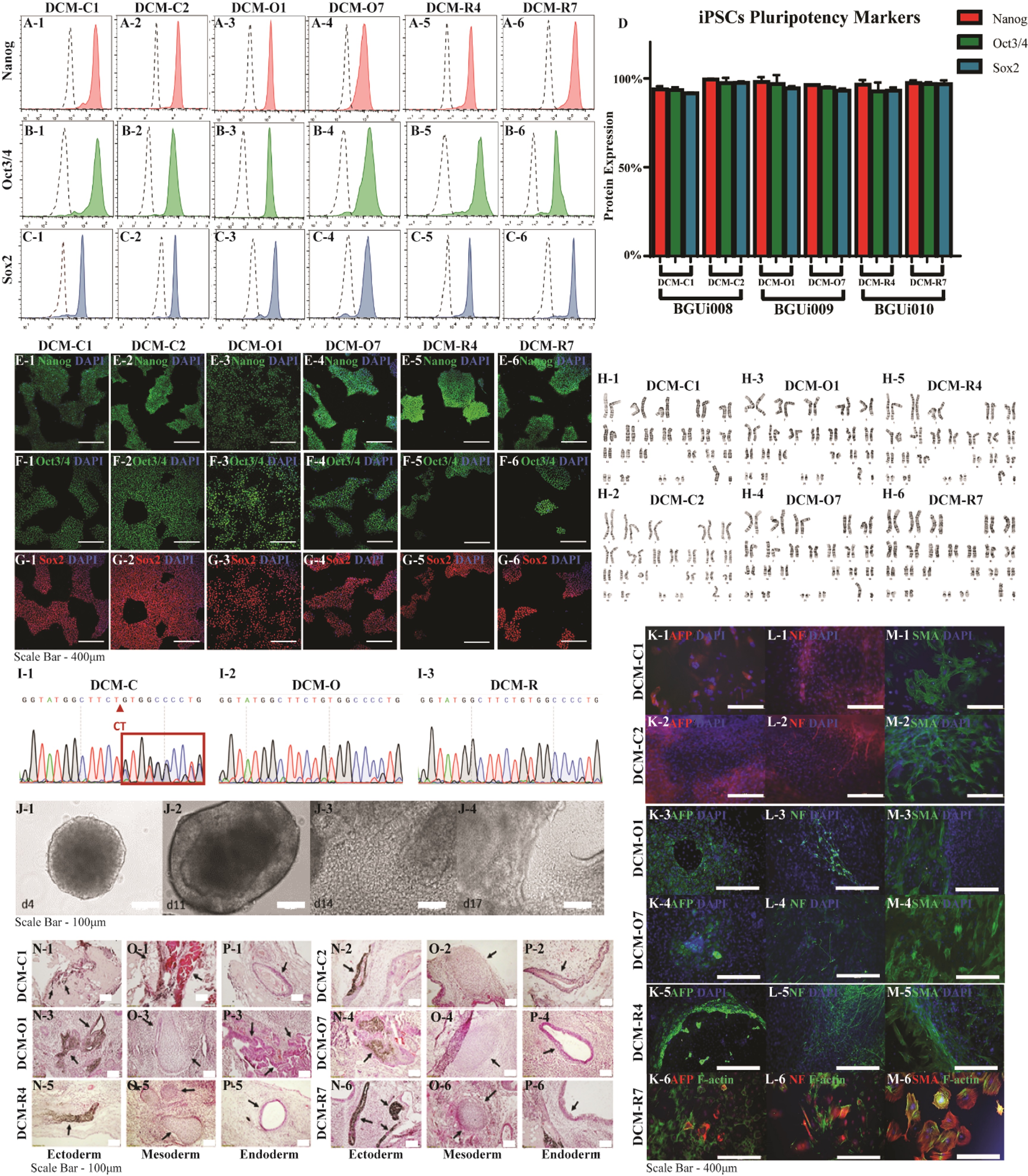

Supplement: Supplementary file 1 — Figure S1: iPSC characterization. Quantitative flow cytometry and immunofluorescence analysis were applied using anti NANOG (A, D, E), Oct ¾ (B, D, F) and Sox2 (C, D, G) antibodies for all iPSC lines. Representative images of karyotype analysis in DCM cell lines (H). DNA sequencing shows the complement sequence of mutation 2156_2157delAG in PLEKHM2 gene in DCM-iPSC lines (I). Spontaneous differentiation steps by EB formation using the hanging drop method (J). All EBs were stained by anti-SMA, anti-NF68, and ant- AFP antibodies (K-M). In vivo teratoma assay of the DCM-iPSCs. Scale bar, 100 μm (N-P). [file 40659_2023_442_MOESM1_ESM.jpg]
